# Infarct morphology assessment in patients with carotid artery/middle cerebral artery occlusion using fast fluid-attenuated inversion recovery (FLAIR) vascular hyperintensity (FVH)

**DOI:** 10.1371/journal.pone.0188078

**Published:** 2017-11-30

**Authors:** Deng-Yue Zhai, Shuang-Gen Zhu, Wei Zhang, Xue Li, You-Ling Zhu

**Affiliations:** 1 Department of Neurology, the Third Affiliated Hospital of Anhui Medical University, Hefei, Anhui Province, China; 2 Department of Neurology, People’s Hospital of Longhua, Shenzhen, Guangdong Province, People’s Republic China; 3 Department of Neurology, the Second People’s Hospital of Anhui Province, Hefei, Anhui Province, China; Henry Ford Health System, UNITED STATES

## Abstract

We aim to evaluate the value of fast fluid-attenuated inversion recovery (FLAIR) vascular hyperintensity (FVH) in assessing infarct morphology in patients with symptomatic internal carotid artery (ICA) or middle cerebral artery (MCA) occlusions. Magnetic resonance (MR) diffusion-weighted imaging (DWI) FLAIR sequences, and carotid/cerebral magnetic resonance angiography of 102 patients with symptomatic ICA or MCA occlusions were evaluated. The location and score of FVH were determined using Olindo’s method; patients were classified as having Low or High FVHs based on FVH score, and either Distal or Proximal FVH based on FVH location. The differences between infarct morphologies were analyzed. FVH were detectable in 62 patients with High FVH and in 40 patients with Low FVHs based on the Olindo’s scale. There were no statistically significant differences in age, gender, hypertension, diabetes, hyperlipidemia, smoking history, and vascular occlusive site between High and Low FVHs patients, except for infarct morphology (*P*<0.01). Patients with Distal FVH presented with significant (*P*<0.01) perforating artery and border zone infarcts, whereas those with Proximal FVH had significant (*P*<0.01) large territorial infarcts. The scores and locations of FVH could be a predictive imaging marker for infarct morphology in patients with symptomatic ICA or MCA occlusion.

## Introduction

Cerebral collateral circulations can be classified as the extra cranial-intracranial collateral circulation, Willis’s cycle, and leptomeningeal collateral circulation. Good collateral circulation can restrain the infarct range of ischemic strokes, and predict the clinical manifestations and prognoses [1] of patients with an infarct. Miteff *et al*. [2] considered good collateral circulation as a useful indicator of excellent prognosis of thrombolytic therapy. Huang *et al* [3] found that good collateral circulation had a positive effect on the clinical manifestations and prognosis of patients with acute cerebral infarcts; and it may provide an evaluation of the benefits of early thrombolytic therapy in those patients. Currently, cerebral angiography has been the gold standard for the assessment of intracranial collateral circulation; however it has not been widely applied due to its disadvantages such as invasiveness and high cost. The pathogenesis of these lesions may be a result of the distal slow and disordered blood flow of the occlusive vessels. Fast fluid-attenuated inversion recovery (FLAIR) is a pulse sequence that is used in magnetic resonance imaging (MRI). It is an inversion recovery technique and can be used for brain imaging to bring out the periventricular hypertensive lesions such as those seen in patients with multiple sclerosis, infarction, subarachnoid hemorrhage, and many more [4–6]. FLAIR vascular hyperintensity (FVH) [7] is an abnormal hyperintense vascular shadow found on MRI in patients with ischemic strokes. Some recent studies [3, 8] have verified the association between FVH and severe vascular stenosis or occlusion. Furthermore, FLAIR functions on the basis of a T2-weighted sequence and cerebrospinal fluid (CSF) signal suppression is enabled for better lesion perceptibility in brain parenchyma when there is direct contact of CSF space with the infarct zone. Additionally, slow flow and immobility result in a high-pitched signal on FLAIR in comparison to the normal ‘flow void’ occurrence of arteries [9].

A distinction between proximal (internal carotid artery, ICA, or M1-middle cerebral artery, M1-MCA) hyperintensities and distal (Sylvian fissure or cortical surface) FVH is essential because it is assumed that proximal vessel signs in the MCA territory represent a thrombus, whereas distal FVH represents slow blood flow [10, 11]. The incidence of FVH within the 3 hours after the onset of stroke symptoms is about 66–77% in the acute stroke patient population [12, 13]. However, the correlation of FVH to infarct size or clinical outcomes is controversial [12, 14].

FVH could indicate the formation of leptomeningeal collateral circulation and serve as a prognostic marker for patients with cerebral infarct. However, there are no studies analyzing the association between FVH and infarct morphology.

Animal stroke research models are an essential component for the evaluation, evolution, understanding mechanisms, diagnostics, and therapeutic outcomes in the study of cerebral ischemia. However, with the exception of a few pig studies, animal data is lacking [15–18]. No new studies are available to magnify the MRI data. The primary objective of this study was to explore the relationship between FVH and infarct morphology in patients with symptomatic internal carotid artery (ICA) or middle cerebral artery (MCA) occlusions.

## Materials and methods

### Patients

This was a retrospective study of a consecutive series of patients admitted with symptomatic ICA or MCA occlusions between July 2013 and July 2014 at The Department of Neurology, The First People’s Hospital of Hefei.

The study protocol was approved by the Ethics Committees of the First People’s Hospital of Hefei and all participants provided written informed consent. The authors had access to data identifying the subjects, but the dataset was anonymized after completion of data collection and for statistical analysis.

Patients were included if they had: (1) an acute anterior circulation infarction within 1 week of the onset of symptoms, (2) large-artery atherosclerotic thrombosis based on the Trial of ORG 10172 in Acute Stroke Treatment (TOAST), (3) completed cerebral MRI inspections, including the diffusion-weighted imaging (DWI) and FLAIR sequences, carotid three-dimensional contrast-enhanced magnetic resonance angiography (3D CE-MRA) and cerebral magnetic resonance angiography (MRA), (4) carotid or cerebral MRA indicated ICA or MCA occlusion, i.e., stenosis = 100%, and (5) received general treatments of anti-platelet aggregation, scavenging free radicals, lipid-lowering with statins and risk factors control.

Patients were excluded if they had: (1) severe posterior circulation stenosis or occlusion, (2) cardiac, cerebral infarction, (3) other reasons, such as Moyamoya disease, vasculitis, hypercoagulable states, as well as unexplained cerebral infarction, (4) failed to complete the MRI examination due to implantation of pacemakers or foreign metallic material inside the body, and/or (5) underwent ultra-early thrombolytic treatment for the arteries and veins.

Patients’ general information (age and sex), history (hypertension, diabetes, hyperlipidemia, smoking history), and imaging data (cerebral DWI and FLAIR sequences, and cerebral/carotid MRA) were collected. The relevant ethics committee approved the protocol.

### MRI protocol

A Siemens MAGNETOM AVANTO 1.5T MRI scanner (Siemens AG, Berlin, Germany) was used for this study. After enrollment, patients received the routine and standardized MRI examinations such as plain scan, DWI, FLAIR, and carotid/cerebral MRA. The main parameters were as follows. T_1_WI: TR = 2 113 ms, TE = 25.2 ms, thickness = 5 mm; T_2_WI: TR = 4 000 ms, TE = 102 ms, thickness = 5 mm; DWI: TR = 6 239 ms, TE = 77.5 ms, thickness = 5 mm; FLAIR: fast spin echo (FSE) sequence, TR = 7 277 ms, TE = 135 ms, thickness = 5 mm; MRA (with the 3D time-of-flight method): TR = 33 ms, TE = 3.8 ms, thickness = 1.2 mm, application of 3D reconstruction using the maximum intensity projection.

### FVH scale

FVH is defined as linear, point-like, or tubular hyperintensity on cerebral MR FLAIR sequence that distributed along the sulci or cerebral surface [19]. The FVH scale was calculated according to the method described by Olindo et al. [20]. Horizontal admission FLAIR MRIs were analyzed from the first M1-MCA appearance to the 10th image. The absence of FVH on one slice was rated as 0 points. One or more FVHs recognized on one slice were rated as 1 point. As 10 images were analyzed, the resulting FVHs ranged from 0 to 10 points. Patients were classified as Low FVHs (FVHs <4, including FVH = 0) or as High FVHs (FVHs ≥ 4).

### FVH location

Patients were defined into the Distal FVH and Proximal FVH based on the anatomical location and distribution of FVH on the FLAIR sequence. The proximal FVH was defined as FVH that was distributed at the MCA M1 or M2 segments while the distal FVH was defined as FVH that distributed at the MCA M3 or distal segments. Since some researchers thought that the pathogenesis of proximal FVH is due to proximal blood stasis of the occlusive vessels, while that of distal FVH is because of the slow blood flow of the distal circulation of the occlusive vessels, patients without FVH or with proximal FVH were included in the proximal FVH cohort while those with distal FVH, accompanied by proximal FVH or not, were placed in the Distal FVH [12] cohort.

### Infarct morphology analysis

Infarct morphology patterns were classified as either single or multiple patterns according to the numbers of the infarct foci on the MR DWI sequence [21]. Based on the MCA region, single cerebral infarcts were classified as perforating artery infarcts, subcortical infarcts, border-zone (BZ) infarcts, and large territorial infarcts, out of which the perforating artery infarcts were further divided into lacunar infarction (φ ≤2 cm) and striatocapsular infarcts (φ >2 cm), whereas the BZ infarcts were further grouped as anterior cortical (territory between the anterior cerebral artery and the middle cerebral artery), posterior cortical (area between the posterior cerebral artery and the middle cerebral artery), or subcortical (territory between cortex branch and the perforating branch) BZ infarcts. Multiple cerebral infarcts were defined as the scattered distribution of hypertensities on the DWI sequence; however, a multiple BZ infarcts referred to those lesions with no less than 2 different vascular territories in the anterior cortical, posterior cortical or subcortical regions simultaneously.

**Fig 1:**
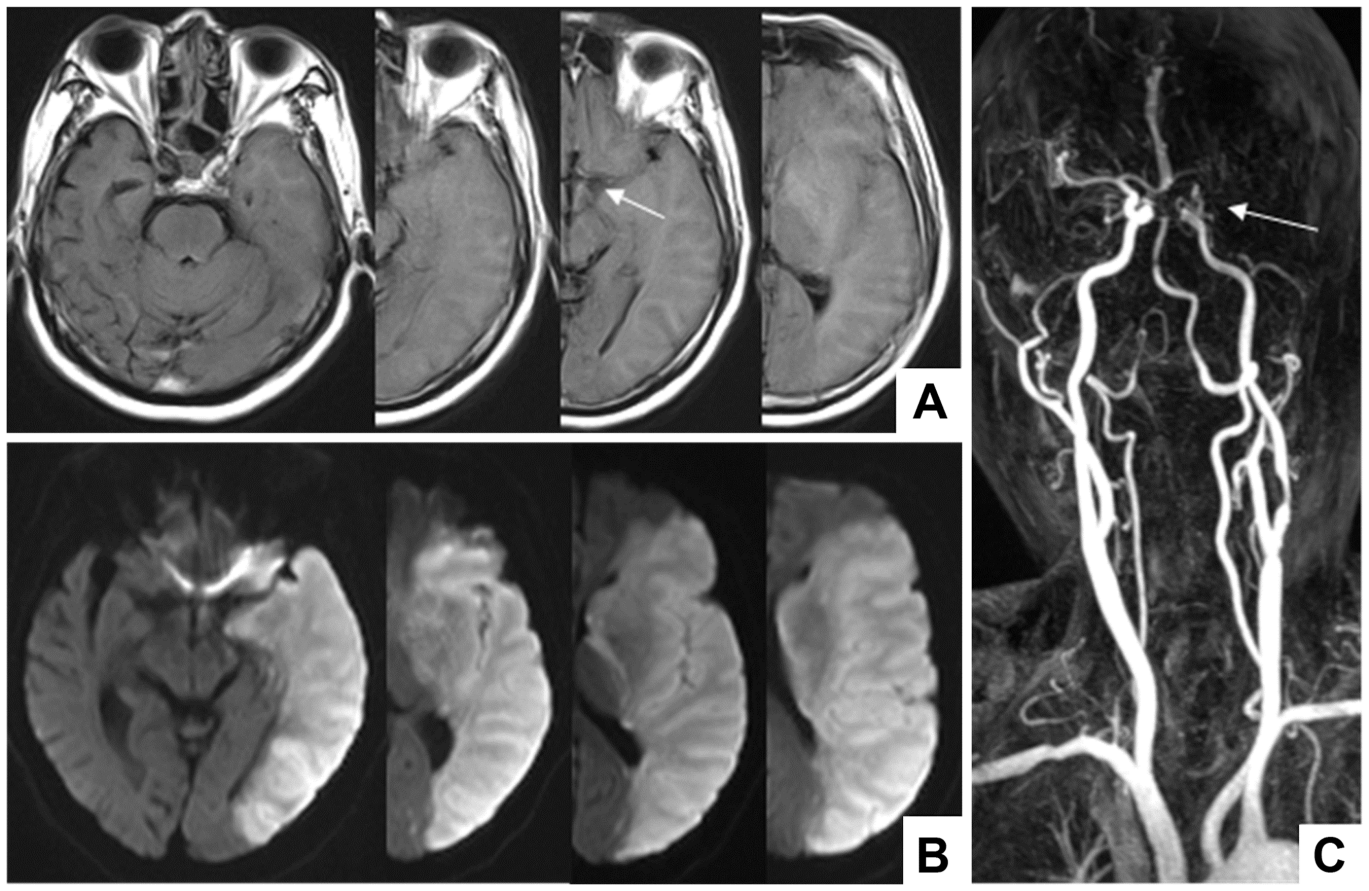
A patient with carotid artery occlusion. A: FVH was found in one image of the FLAIR sequence, that was located at the proximal segment; therefore, this patient was defined in the Low FVHs patients and Proximal FVH patients respectively; B: DWI sequence indicated a massive cerebral infarction of the left hemisphere; C: carotid MRA showed distal occlusion of the left internal carotid artery.

**Fig 2:**
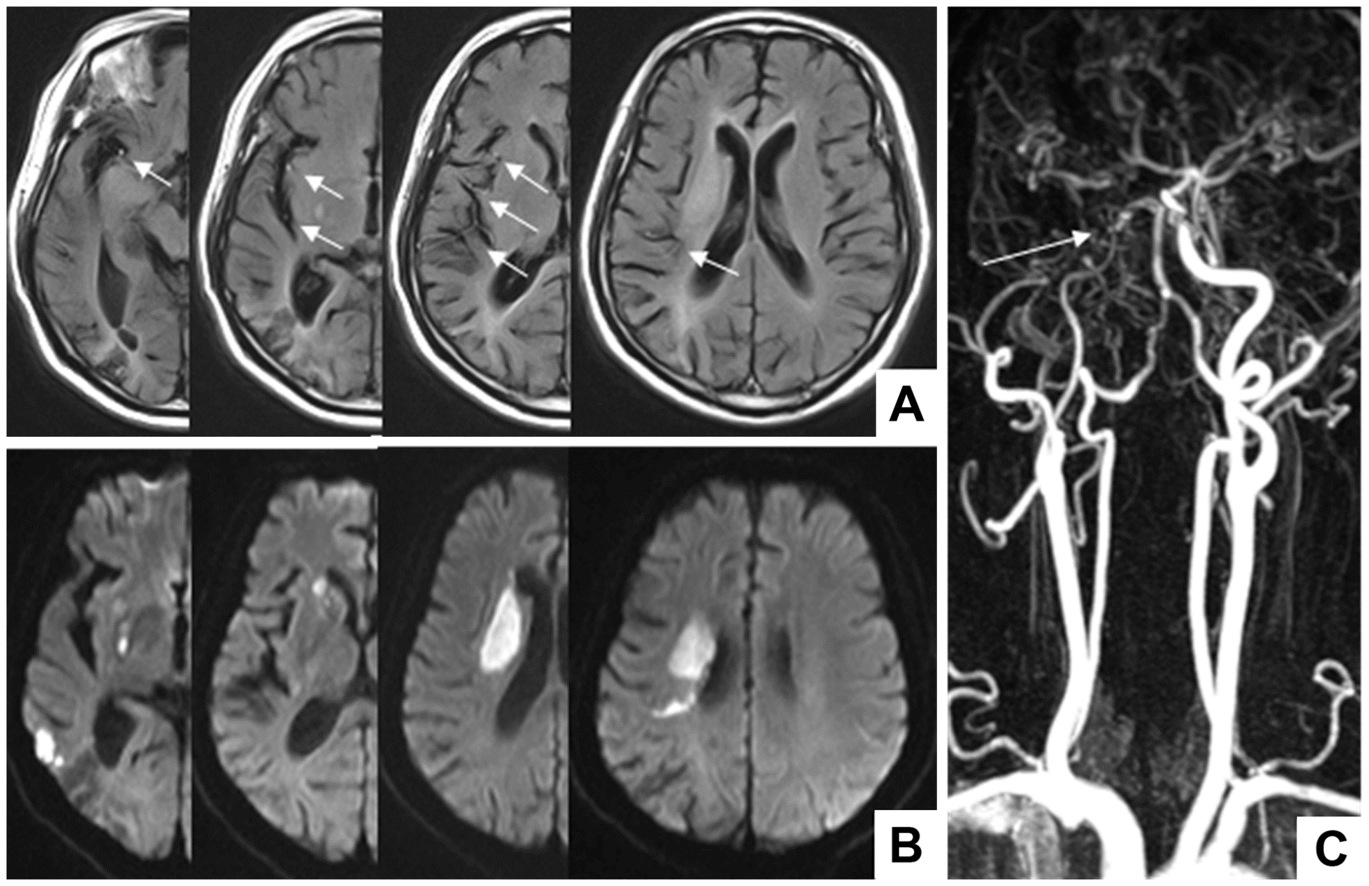
A patient with carotid artery occlusion. A: FVH was found in more than 4 images in the FLAIR sequence, which were located both at the proximal and distal segments; therefore, this patient was defined as the High FVHs and Distal FVH, respectively; B: DWI sequence indicated perforating artery infarct + cortical branch infarct; C: carotid MRA showed distal occlusion of the right internal carotid artery.

Two experienced senior deputy chief physicians of neurology blinded to the other sequences quantified the FVH. The reading contents included degrees of vascular stenosis, FVH assessments, and infarct morphology. When a dispute appeared, another neurological deputy chief was invited to reach a consensus. Figs 1 and 2 show the examples of FVH definition and infarct morphology.

### Statistical analysis

SPSS) 16.0 (IBM, Armonk, NY, USA) was used to perform the statistical analysis. Descriptive analysis was used to express the demographic data, out of which the normally distributed measurement data were expressed as mean ± standard derivation (SD), skewed data were expressed as the median and interquartile range, and the enumeration data were expressed as percentages. The normally distributed data were analyzed by the independent samples t-test, skewed data were analyzed by the non-parametric test, and the enumeration data were analyzed by the *χ*^*2*^ test. P values of less than 0.05 were considered statistically significant.

## Results

### Comparison of the baseline data among the patients from each group

A total of 102 patients [54 (52.9%) men, and 48 (47.1%) women] met the inclusion criteria; their ages ranged between 44 to 86 years old, with a median age of 73. Of these 102 patients, 40 (39.2%) had Low FVHs and 62 (60.8%) had High FVHs. No significant differences were found amongst the general clinical data relating to age, sex, hypertension, and diabetes, hyperlipidemia, smoking history or vascular occlusion site between these two groups (*P*>0.05) (Table 1).

**Table 1:** Comparison of the demographics, general clinical data between the Low FVHs and High FVHs groups [*n* (%)].

Item	Low FVHs	High FVHs	*χ*^*2*^ or *t* value	*P* value
	(*n* = 40)	(*n* = 62)		
Age (years, mean ± SD)	70.3±10.8	71.4±10.9	0.385	0.704
Sex-male/female	18 (45.0)/22 (55.0)	36 (58.1)/26 (41.9)	1.666	0.197
Hypertension	22 (55.0)	42 (67.7)	1.689	0.194
Diabetes	10 (25.0)	22 (35.5)	1.241	0.265
Hyperlipidemia	12 (30.0)	24 (38.7)	0.808	0.369
Smoking history	2 (5.0)	6 (9.7)	0.736	0.391
Vascular occlusion site				
Internal carotid artery	14 (35.0)	22 (35.5)	0.13	0.937
MCA M1 segment	18 (45.0)	26 (41.9)		
M2 segment of middle cerebral artery	8 (20.0)	14 (22.6)		

### Infarct morphology between Low FVHs and High FVHs patients

**Fig 3:**
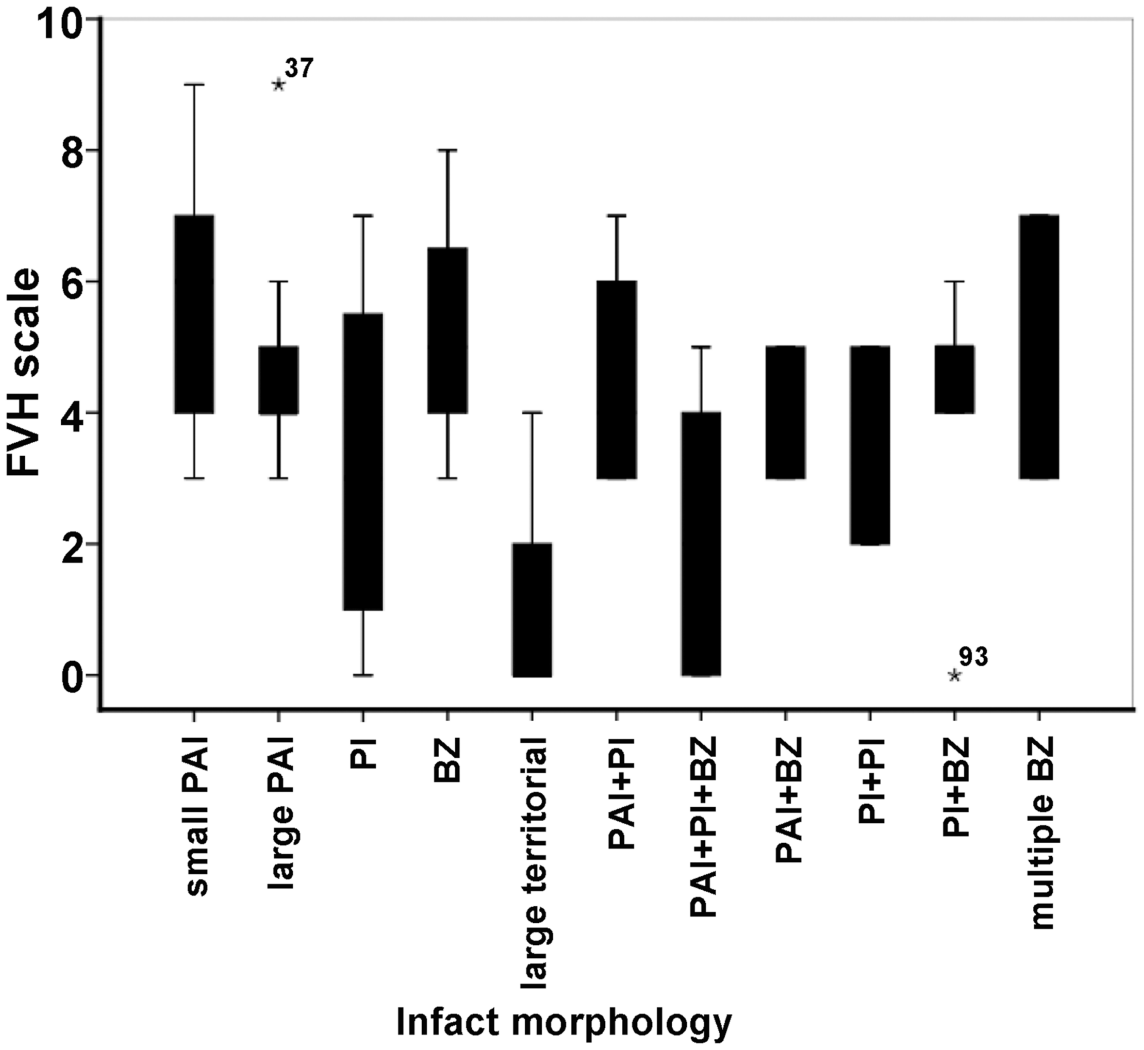
Box plots of the FVH scale in relation to infarct morphology.

The perforating artery infarct was the most frequently occurring lesion in the High FVHs patients (*n* = 32, 51.6%), followed by BZ infarct (*n* = 8, 12.9%), and one case (1.6%) of large territorial infarct. Conversely in the Low FVHs patients, a large territorial infarct was the most common lesion (*n* = 17, 42.5%, *P*<0.01, Table 2). Fig 3 presents the FVH scale in relation to infarct morphology.

**Table 2:** Comparison of various infarct morphology between the Low FVHs and High FVHs patients [*n* (%)].

Item	Low FVHs	High FVHs	*χ*^*2*^	*P* value
	(*n* = 40)	(*n* = 62)		
Single pattern			38.917	<0.001
Perforating artery infarct (φ ≤2 cm)	2 (5.0)	18 (29.0)		
Perforating artery infarct (φ >2 cm)	3 (7.5)	14 (22.6)		
Cortical branch infarct	2 (5.0)	2 (3.2)		
BZ infarct	3 (7.5)	8 (12.9)		
Large territorial infarct	17 (42.5)	1 (1.6)		
Multiple pattern				
Perforating artery infarct + Cortical branch infarct	2 (5.0)	4 (6.5)		
Perforating artery infarct + Cortical branch infarct + BZ infarct	4 (10.0)	2 (3.2)		
Perforating artery infarct + BZ infarct	2 (5.0)	4 (6.5)		
Multiple cortical branch infarcts	2 (5.0)	2 (3.2)		
Cortical branch infarct + BZ infarct	1 (2.5)	4 (6.5)		
Multiple BZ infarcts	2 (5.0)	3 (4.8)		

### Infarct morphology in various FVH location

Among the 102 patients in the study, 26 (25.5%) were in the Proximal FVH cohort while 76 (74.5%) were in the distal FVH cohort. Comparison of the infarct morphology between Proximal FVH and Distal FVH patients indicated that perforating artery infarct was the most frequently observed lesion in Distal FVH patients (*n* = 37, 48.7%), followed by BZ infarct (*n* = 11, 14.5%), while the cortical branch infarct (*n* = 2, 2.6%) and large territorial infarct (*n* = 2, 2.6%) were rare. Conversely, in Proximal FVH patients, large territorial infarct was the most commonly found lesion (*n* = 16, 61.5%), and the remaining were mostly multiple lesions (*n* = 8, 30.8%*P*< 0.01, Table 3).

**Table 3:** Comparison of various infarct morphology in patients with FVH observed at different infarct sites [*n* (%)].

Item	Proximal FVH	Distal FVH	*χ*^*2*^ value	*P* value
	(*n* = 26)	(*n* = 76)		
Single pattern			62.285	<0.001
Perforating artery infarct (φ ≤2 cm)	0	20 (26.3)		
Perforating artery infarct (φ >2 cm)	0	17 (22.4)		
Cortical branch infarct	2 (7.7)	2 (2.6)		
BZ infarct	0	11 (14.5)		
Large territorial infarct	16 (61.5)	2 (2.6)		
Multiple pattern				
Perforating artery infarct + Cortical branch infarct	0	6 (7.9)		
Perforating artery infarct + Cortical branch infarct + BZ infarct	4 (15.4)	2 (2.6)		
Perforating artery infarct + BZ infarct	0	6 (7.9)		
Multiple cortical branch infarcts	2 (7.7)	2 (2.6)		
Cortical branch infarct + BZ infarct	1 (3.8)	4 (5.3)		
Multiple BZ infarcts	1 (3.8)	4 (5.3)		

## Discussion

With the advancement in neuroimaging technology, applications of FVH in cerebral ischemic diseases have drawn a widespread interest amongst researchers. Our study used patients with symptomatic internal carotid artery (ICA) or middle cerebral artery (MCA) occlusions and found that FVH was predictive of infarct morphology. The results of the present study provide new data about the relations among vascular occlusion, low perfusion, advantages and disadvantages of collateral circulation, and distribution patterns of infarction lesions. As highlighted by Kim et al. [22], FVH may help identifying patients with CBZ infarct who may require closer observation and hemodynamic control. Indeed, the exact underlying mechanism of stroke is an important determinant of the prognosis. For now, this tool should be used in conjunction with other tools as it is not yet validated in large multicenter trials. FVH could also eventually be used for the diagnosis of intracranial large artery stenosis and occlusion diseases such as atherosclerosis, Moyamoya disease, etc.

The association between FVH and infarct size and clinical outcomes is controversial [12, 14]. In the setting of acute arterial occlusion, high blood vessel signal recovered by FVH was correlated with slow blood flow of leptomeningeal collateral and low cerebral perfusion. Some authors showed that the presence of FVH was associated with larger infarct volumes and poorer clinical outcomes [14], while other studies showed the contrary [3, 12, 20]. The present study showed that the presence of FVH in the distal end of the artery occlusion was associated with better prognosis and smaller infarct size. Different FVH scoring method could explain some differences among studies. Indeed, Lee et al. [12] described distal FVH as occurring at the end of occlusion or stenosis. The large number of distal FVH reflected well compensated leptomeningeal collateral flow, resulting in a smaller infarct size and a lower initial NIHSS score. In the same way, the scoring method by Olindo et al. [20] also focused on the quantification of the distal FVH of the middle cerebral artery occlusion. On the other hand, Hohenhaus et al. [14] used the ASPECTS scale for FVH scoring and this scale only scores two levels of the middle cerebral artery, which could lead to deficiencies in the quantification of distal FVH. Recently, Liu et al. [23] suggested that higher FVH-ASPECTS measured outside the DWI lesion was associated with good clinical outcomes in patients undergoing ET. FVH-ASPECTS measured inside the DWI lesion was predictive of hemorrhagic transformation [23]. The FVH pattern, not number, could serve as an imaging selection marker for ET in acute middle cerebral artery occlusion [23]. In addition, the above studies were all single center and the small sample size, ethnic, and regional differences could also lead to discrepancies, pending for further multicenter studies.

Our study indicated that perforating artery infarcts (51.6%) and border-zone (BZ) infarcts (12.9%) were the most frequently types of infarcts found in patients with higher FVH score, whereas large territorial infarcts (1.6%) were rare in these patients. While significant territorial infarcts (42.5%) were the most commonly found lesions in the patients with lower FVH scores, and perforating artery infarcts (12.5%), however, BZ infarcts (7.5%) were less frequently observed. Compared with the patients with lower FVH scores, the infarct foci in the high FVH score patients were mostly located around the striatocapsular and border-zone areas and they were in small sizes, which was possibly associated with better leptomeningeal collateral circulation in the high FVH score patients [9]. As a significant cerebral circulation, leptomeningeal collateral circulation increases the reperfusion of the ischemic regions and rescues the ischemic brain tissues. Although patients in this study had ICA or MCA occlusions, patients with FVH had less damaged brain tissue compared with those without FVH, and this difference became more significant as the FVH score increased.

Distal FVH [24] refers to the hyperintense shadows of tiny serpiginous vascular-like structures in the Sylvian fissure extending distally through the convoluted architecture of the cerebral sulci. This hyperintensity may result from the distal-to-proximal compensatory reflux of the stenotic or occlusive vessels, since the slow blood flow will lead to a disappearance of the flowing void effect and lead to hyperintensity, serving as a marker of the leptomeningeal collateral circulation. Proximal FVH [3] refers to the punctate and cord-like hyperintensities that were located at the sites or the proximal segment of the stenotic and occlusive vessels, serving as a marker for the severe intracranial arterial stenosis or occlusion. Our results found that perforating artery infarcts (48.7%) and BZ infarcts (14.5%) were the most frequent infarcts in patients with a distal FVH, while large territorial infarcts (61.5%) were the most common infarcts in the proximal FVH patients. One reason for this difference may be explained by the different pathogeneses in the formation of these two FVH patterns. A better leptomeningeal collateral circulation, more frequent infarct location at the striatocapsular and border-zone areas, and few infarcts (2.6%) in the cortical region were found in the distal-FVH patients, while an inadequately formed collateral circulation and the most common large territorial infarcts (61.5%) were observed in the proximal FVH patients. The distal FVH represented the leptomeningeal collateral circulation in the distal segments of the occlusive vessels while the proximal FVH could only represent the occlusive vessels, which was also consistent with the study of Liebeshind *et al*. [24].

Based on their anatomical characteristics, cerebral collateral circulations can be classified as extra cranial-intracranial collateral circulation, Willis’s cycle, and leptomeningeal collateral circulation. Good collateral circulation can restrain the infarct range of the ischemic strokes, and presents particular assessed values in predicting the clinical manifestations and prognoses [1]. Miteff *et al*. [2] considered good collateral circulation as a useful indicator of excellent prognosis of thrombolytic therapy. Huang *et al* [3] found that good collateral circulation had a positive effect on clinical manifestations and prognoses of the patients with acute cerebral infarcts, and could provide an evaluation of the benefits of early thrombolytic therapy in those patients. At present, cerebral angiography has been the gold standard for the assessment of intracranial collateral circulation; however it has not been widely applied due to its disadvantages such as invasiveness and high cost.

This study also found that infarct morphology differed in patients with various FVH numbers and different FVH locations. The possible reason for this might be the differences in the leptomeningeal collateral circulations. Therefore, assessment of collateral circulation in patients with acute cerebral infarcts should be conducted according to the FVH number and site on the FLAIR sequence MRI, infarct morphology on the DWI sequence, and the associated vascular examinations. After that, an individualized treatment regimen could be planned after a further evaluation of the clinical conditions and prognosis, thus providing a simple, convenient and economical radiographic method for evaluating the intracranial collateral circulations, in particularly the leptomeningeal collateral circulation.

Some of the limitations of this study include the relatively aged patient population which can affect some of the parameters evaluated in the study. Moreover, only ICA or MCA patients were included in this study. It was a single center trial performed in elderly patients (median age of 73 years). This study focused only on the morphology of infarct without evaluation of the clinical outcomes. The patients did not undergo MRI perfusion weighted image examination, and correlations among different parameters could be explored. Therefore, large, multi-center randomized control trials are needed to provide additional evidence.

## Conclusions

In summary, this study showed that FVH at different locations and various numbers had was predictive of infarct morphology. Moreover, if combined with the associated radiographic and vascular examinations, FVH may assess the collateral circulations in patients with acute cerebral infarcts, serving as a prognostic indicator.

## Supporting information

S1 Data Raw data. (XLSX)

## Abbreviations

BZ: Border zone
DWI: diffusion-weighted imaging
FLAIR: fluid-attenuated inversion recovery
FVH: vascular hyperintensity
ICA: internal carotid artery
MCA: middle cerebral artery
MR: Magnetic resonance
MRA: magnetic resonance angiography

## References

[1] Shuaib A, Butcher K, Mohammad AA, Saqqur M, Liebeskind DS. Collateral blood vessels in acute ischaemic stroke: a potential therapeutic target. Lancet Neurol. 2011; 10: 909–921. doi: 10.1016/S1474-4422(11)70195-8 21939900 10.1016/S1474-4422(11)70195-8

[2] Miteff F, Levi CR, Bateman GA, Spratt N, McElduff P, Parsons MW. The independent predictive utility of computed tomography angiographic collateral status in acute ischaemic stroke. Brain. 2009; 132: 2231–2238. doi: 10.1093/brain/awp155 19509116 10.1093/brain/awp155

[3] Huang X, Liu W, Zhu W, Ni G, Sun W, Ma M, et al Distal hyperintense vessels on FLAIR: a prognostic indicator of acute ischemic stroke. Eur Neurol. 2012; 68: 214–220. doi: 10.1159/000340021 22964844 10.1159/000340021

[4] Bakshi R, Ariyaratana S, Benedict RH, Jacobs L. Fluid-attenuated inversion recovery magnetic resonance imaging detects cortical and juxtacortical multiple sclerosis lesions. Arch Neurol. 2001; 58: 742–748. 11346369 10.1001/archneur.58.5.742

[5] Okuda T, Korogi Y, Ikushima I, Murakami R, Nakashima K, Yasunaga T, et al Use of fluid-attenuated inversion recovery (FLAIR) pulse sequences in perinatal hypoxic-ischaemic encephalopathy. Br J Radiol. 1998; 71: 282–290. doi: 10.1259/bjr.71.843.9616237 9616237 10.1259/bjr.71.843.9616237

[6] Bangerter NK, Hargreaves BA, Gold GE, Stucker DT, Nishimura DG. Fluid-attenuated inversion-recovery SSFP imaging. J Magn Reson Imaging. 2006; 24: 1426–1431. doi: 10.1002/jmri.20743 17036358 10.1002/jmri.20743

[7] Kim SJ, Ha YS, Ryoo S, Noh HJ, Ha SY, Bang OY, et al Sulcal effacement on fluid attenuation inversion recovery magnetic resonance imaging in hyperacute stroke: association with collateral flow and clinical outcomes. Stroke. 2012; 43: 386–392. doi: 10.1161/STROKEAHA.111.638106 22096035 10.1161/STROKEAHA.111.638106

[8] Kawashima M, Noguchi T, Takase Y, Nakahara Y, Matsushima T. Decrease in leptomeningeal ivy sign on fluid-attenuated inversion recovery images after cerebral revascularization in patients with Moyamoya disease. AJNR Am J Neuroradiol. 2010; 31: 1713–1718. doi: 10.3174/ajnr.A2124 20466798 10.3174/ajnr.A2124 PMC7964983

[9] Sanossian N, Saver JL, Alger JR, Kim D, Duckwiler GR, Jahan R, et al Angiography reveals that fluid-attenuated inversion recovery vascular hyperintensities are due to slow flow, not thrombus. AJNR Am J Neuroradiol. 2009; 30: 564–568. doi: 10.3174/ajnr.A1388 19022866 10.3174/ajnr.A1388 PMC2729168

[10] Toyoda K, Ida M, Fukuda K. Fluid-attenuated inversion recovery intraarterial signal: an early sign of hyperacute cerebral ischemia. AJNR Am J Neuroradiol. 2001; 22: 1021–1029. 11415892 PMC7974782

[11] Assouline E, Benziane K, Reizine D, Guichard JP, Pico F, Merland JJ, et al Intra-arterial thrombus visualized on T2* gradient echo imaging in acute ischemic stroke. Cerebrovasc Dis. 2005; 20: 6–11. doi: 10.1159/000086120 15925876 10.1159/000086120

[12] Lee KY, Latour LL, Luby M, Hsia AW, Merino JG, Warach S. Distal hyperintense vessels on FLAIR: an MRI marker for collateral circulation in acute stroke? Neurology. 2009; 72: 1134–1139. doi: 10.1212/01.wnl.0000345360.80382.69 19211928 10.1212/01.wnl.0000345360.80382.69 PMC2677466

[13] Schellinger PD, Chalela JA, Kang DW, Latour LL, Warach S. Diagnostic and prognostic value of early MR Imaging vessel signs in hyperacute stroke patients imaged <3 hours and treated with recombinant tissue plasminogen activator. AJNR Am J Neuroradiol. 2005; 26: 618–624. 15764589 PMC7976462

[14] Hohenhaus M, Schmidt WU, Brunecker P, Xu C, Hotter B, Rozanski M, et al FLAIR vascular hyperintensities in acute ICA and MCA infarction: a marker for mismatch and stroke severity? Cerebrovasc Dis. 2012; 34: 63–69. doi: 10.1159/000339012 22759720 10.1159/000339012

[15] Shi ZQ, Sunico CR, McKercher SR, Cui J, Feng GS, Nakamura T, et al S-nitrosylated SHP-2 contributes to NMDA receptor-mediated excitotoxicity in acute ischemic stroke. Proc Natl Acad Sci U S A. 2013; 110: 3137–3142. doi: 10.1073/pnas.1215501110 23382182 10.1073/pnas.1215501110 PMC3581884

[16] McNally JS, Kim SE, Yoon HC, Findeiss LK, Roberts JA, Nightingale DR, et al Carotid magnetization-prepared rapid acquisition with gradient-echo signal is associated with acute territorial cerebral ischemic events detected by diffusion-weighted MRI. Circ Cardiovasc Imaging. 2012; 5: 376–382. doi: 10.1161/CIRCIMAGING.111.967398 22495769 10.1161/CIRCIMAGING.111.967398

[17] Machado LS, Kozak A, Ergul A, Hess DC, Borlongan CV, Fagan SC. Delayed minocycline inhibits ischemia-activated matrix metalloproteinases 2 and 9 after experimental stroke. BMC Neurosci. 2006; 7: 56 doi: 10.1186/1471-2202-7-56 16846501 10.1186/1471-2202-7-56 PMC1543649

[18] Hill WD, Hess DC, Martin-Studdard A, Carothers JJ, Zheng J, Hale D, et al SDF-1 (CXCL12) is upregulated in the ischemic penumbra following stroke: association with bone marrow cell homing to injury. J Neuropathol Exp Neurol. 2004; 63: 84–96. 14748564 10.1093/jnen/63.1.84

[19] Chung PW, Park KY. Leptomeningeal enhancement in petients with moyamoya disease: correlation with perfusion imaging. Neurology. 2009; 72: 1872–1873. doi: 10.1212/WNL.0b013e3181a7120e 19470971 10.1212/WNL.0b013e3181a7120e

[20] Olindo S, Chausson N, Joux J, Saint-Vil M, Signate A, Edimonana-Kapute M, et al Fluid-attenuated inversion recovery vascular hyperintensity: an early predictor of clinical outcome in proximal middle cerebral artery occlusion. Arch Neurol. 2012; 69: 1462–1468. doi: 10.1001/archneurol.2012.1310 22893218 10.1001/archneurol.2012.1310

[21] Lee DK, Kim JS, Kwon SU, Yoo SH, Kang DW. Lesion patterns and stroke mechanism in atherosclerotic middle cerebral artery disease: early diffusion-weighted imaging study. Stroke. 2005; 36: 2583–2588. doi: 10.1161/01.STR.0000189999.19948.14 16269637 10.1161/01.STR.0000189999.19948.14

[22] Kim SE, Lee BI, Kim SE, Shin KJ, Park J, Park KM, et al Clinical Significance of Fluid-Attenuated Inversion Recovery Vascular Hyperintensities in Borderzone Infarcts. Stroke. 2016; 47: 1548–1554. doi: 10.1161/STROKEAHA.115.012285 27217507 10.1161/STROKEAHA.115.012285

[23] Liu D, Scalzo F, Rao NM, Hinman JD, Kim D, Ali LK, et al Fluid-Attenuated Inversion Recovery Vascular Hyperintensity Topography, Novel Imaging Marker for Revascularization in Middle Cerebral Artery Occlusion. Stroke. 2016; 47: 2763–2769. doi: 10.1161/STROKEAHA.116.013953 27659851 10.1161/STROKEAHA.116.013953 PMC5079823

[24] Liebeskind DS. Location, location, location: angiography discerns early MR imaging vessel signs due to proximal arterial occlusion and distal collateral flow. AJNR Am J Neuroradiol. 2005; 26: 2432–2433; author reply 2433–2434. 16219865 PMC7976115

